# Committing to Keep Clean: Nudging Complements Standard Policy Measures to Reduce Illegal Urban Garbage Disposal in a Neighborhood With High Levels of Social Cohesion

**DOI:** 10.3389/fpsyg.2021.660410

**Published:** 2021-07-27

**Authors:** Inge Merkelbach, Malte Dewies, Semiha Denktas

**Affiliations:** Erasmus School of Social and Behavioural Sciences, Erasmus University Rotterdam, Rotterdam, Netherlands

**Keywords:** nudging, garbage disposal, field experiment, behavioral insights, Behavioral Insights Group Rotterdam

## Abstract

Illegal garbage disposals are a persistent urban problem, resulting in high clean-up costs, and nuisance and decreased satisfaction with the neighborhood among residents. We compared three adjacent city-areas in Rotterdam in the Netherlands which, for 2 weeks, either: (1) no action to decrease illegal garbage disposals was taken; (2) standard door-to-door canvassing was carried out; or (3) door-to-door canvassing was enriched with several nudges, most importantly a commitment-nudge. The nudge treatment proved highly effective, reducing illegal disposals at post-test and follow-up (2 months later) with two-thirds, resulting in a very large effect size (*d* = 2.60). At post-test, standard door-to-door canvassing did not differ from the control treatment, but at follow-up results were comparable to the nudging-treatment. This could, however, be due to spill-over effects. Using a commitment nudge thus proved highly effective in decreasing illegal garbage disposals, however, effects might be specific to neighborhoods with strong social cohesion.

## Introduction

Illegal garbage disposal is a serious problem in Rotterdam, the second largest city of the Netherlands with approximately 625,000 inhabitants. In a representative survey among the city population (Neighborhood Profile Rotterdam, 2016), 19% named littering as their number one nuisance, compared to 10% on national level. Concerning the consequences of disposing garbage in public, littering was shown to reduce the aesthetical quality of the environment (Roda et al., 2016). Brown and Raymond (2006) showed that residents identify aesthetical quality as very important, and that it was predictive for residents’ place attachment. Place attachment in turn was found to be positively related to life satisfaction, further highlighting the importance of reducing illegal garbage disposal to improve the subjective experiences of Rotterdam residents (Casakin and Reizer, 2017). Moreover, reduced place attachment is related to reduced efforts into caring for one’s residential environment (e.g., Mohapatra and Mohamed, 2013).

Littering is a potentially self-reinforcing problem. Various studies have shown that visible garbage in public spaces invites individuals to litter more in these spaces themselves (e.g., Keizer et al., 2011; Schultz et al., 2013). As an explanation it has been suggested that the presence of garbage indicates the prevailing social norm for how to dispose one’s garbage, i.e., by leaving it in public spaces (Cialdini et al., 1990). Garbage placed next, instead of into, containers is a huge problem.

To prevent the emergence of a vicious littering-cycle as well as to increase residents’ attachment with their neighborhood, in the past the municipality of Rotterdam put substantial effort into keeping public spaces clean. Illegal garbage was for instance frequently picked up by the municipality, up to multiple times a day in the neighborhoods with the most severe littering problems. This was, however, very costly and did not reduce illegal disposals, possibly because it stimulated free riding behavior on public services (Firschbacher and Gachter, 2010). Until now, no effective interventions have been implemented in Rotterdam. A recent experiment in which pick-ups were reduced in an attempt to increase residents’ own responsibility did also not have the desired effect (Dur and Vollaard, 2015), but even resulted in increased littering. Because current practice (i.e., frequent pick-ups) is costly and reduced pick-ups resulted in more littering, we focused on measures beyond pick-up frequency.

Specifically, we aimed to improve the standard canvassing policy of the municipality adding to it a “behavioral spin” (Loer, 2019). Typically, policy measures assume individuals to react to them rationally (Howlett, 2018) meaning that they are designed to target rational thought. In fact, canvassing focused on information about rules for and possible consequences of illegal garbage disposal (e.g., fines, attracting vermin) hoping that information would stimulate behavior change. However, rational-based approaches regularly fall short limiting the effectiveness of policy measures (Weaver, 2015). Therefore, it has been suggested to use behavioral insights to improve effectiveness (Thaler and Sunstein, 2009). A challenge, however, has been how to complement existing measures with behavioral insights (Loer, 2019). Such complementary measures have recently been described as the “most promising frontier” (Ewert, 2020) in behavioral public policy as most applications in the past have treated behavioral measures as standalone solutions (Hansen, 2018; Sanders et al., 2018). Yet, behavioral measures were said to be more effective if taking into account the wider (policy) context (de Ridder et al., 2020). With this study we aim to take a step in that direction.

One of the main challenges to behavior change is the intention-behavior gap (Sheeran and Webb, 2016) where individuals fail to follow-up on their intentions. Assuming that canvassing affected intention but failed to bridge the intention behavior gap, we complemented canvassing using two nudges (Thaler and Sunstein, 2009). Nudges are light touch interventions that require little cognitive engagement from those targeted by the nudges (Thaler and Sunstein, 2009). This is because nudges tend to trigger automatic cognitive processes (e.g., biases and heuristics) in those targeted by the nudges bringing about predictable behavior change in a more subtle way. People are for example always more likely to select the default option, independently of its content (e.g., Van Kleef et al., 2018)^1^. Nudges can be effective for mindless and subconscious behaviors, like littering (French, 2011). However, previous research shows that interventions directed at breaking unconscious behavior and making residents aware of and reflect on the challenges of their neighborhood (e.g., burglary) also have the potential to evoke long lasting behavior changes that benefit the community (Roach et al., 2020). In this study we therefore tried to stimulate both conscious and unconscious processes. The first nudge asked individuals reached by canvassing to show *commitment* to keeping the neighborhood clean by placing a sticker on their doorpost. Commitment nudges have been shown to harbor a large potential in evoking pro-environmental behaviors (e.g., Baca-Motes et al., 2012). Additionally, we expect that the canvassing itself will make people consciously think about the littering challenges in their neighborhood. For the second nudge visual *reminders* were employed that depicted the desired behavior and focused on strengthening a positive sense of *community identity* (i.e., by emphasizing group membership and shared responsibility for the neighborhood; Kolodko et al., 2016). Strengthening community identity has been shown to be effective for evoking pro-environmental behaviors before (Van Vugt, 2009). We assumed an appeal to community identity to be effective because the study area was characterized by high levels of social cohesion, which is associated by increased receptivity to community norms (Forrest and Kearns, 2001). This way, the nudges were integrated with the existing policy structure and city context rather than a standalone approach.

We compared both the standard canvassing policy and the same policy complemented with nudges to a control treatment, in which no actions directed at reducing illegal garbage disposals were carried out. We hypothesized that:

*When comparing the pre-test with the post-test or follow-up, the number of days garbage is illegally disposed would be reduced after carrying out either the standard policy or the standard policy enriched with nudges, with a larger reduction for the nudging treatment.*

## Materials and Methods

### Environmental Context

**FIGURE 1:**
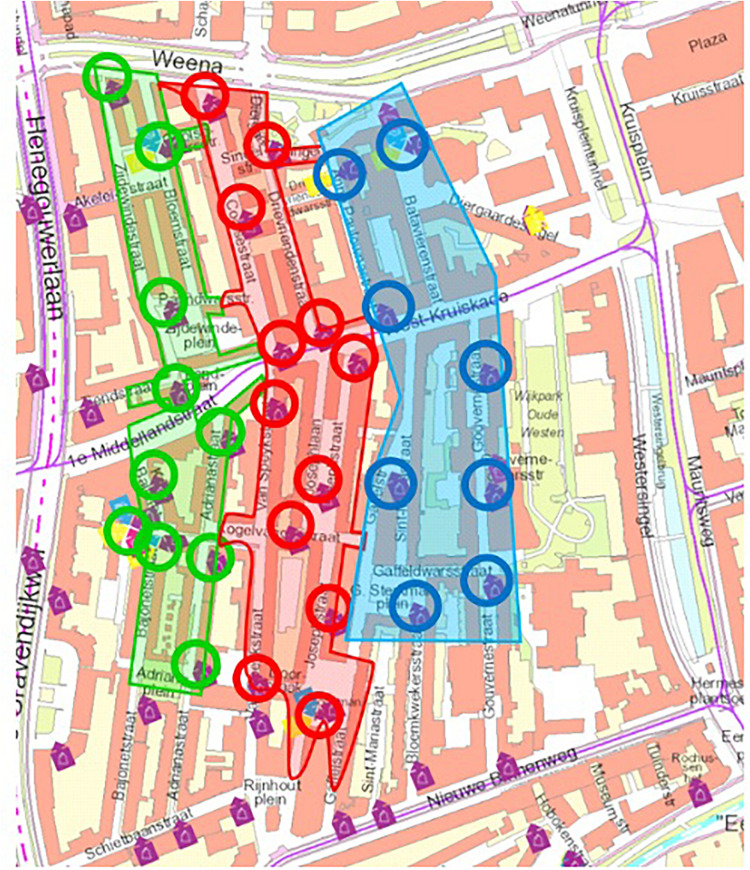
Map of the study area: control treatment = blue, standard treatment = red, nudging treatment = green, circles = container locations.

This study took place in a neighborhood (i.e., Oude Westen) close to the city center of Rotterdam. We made use of a convenience sample in which the control treatment (eight container locations) was carried out in the blue study area, the standard policy treatment (12 container locations) was carried out in the red area, and the nudging treatment (10 containers and seven at follow-up due to road work) was carried out in the green area (Figure 1).

In Rotterdam, placing garbage outside garbage containers was illegal and resulted in a fine between €95 and €500 if detected. Public garbage containers were typically located underground with only a small part of the container being visible. To dispose garbage, residents used a container opening at hip height. Based on the amount of produced garbage, the frequency of emptying containers differed per city area. In the study area, containers were emptied when sensors inside the containers gave a digital signal that the container was almost full. Garbage outside the containers was collected daily, also during the experiment. For disposing items too big for the container openings (i.e., bulky waste) residents needed to arrange free individual pick-up or could bring their bulky waste to depot recycling.

The study area was densely populated (1,100 households) and characterized by high levels of ethnic diversity (71% of the residents had a migration background; compared to 49% on city level; Neighborhood Profile Rotterdam, 2016) and a relatively low mean income (65% of the residents were defined as receiving a “low” income; compared to 51% on city level). The vast majority of residents was between 15 and 65 years old (72%). Residents of the study area reported that illegal littering caused them substantial nuisance: 55% often experienced annoyance due to garbage placed outside containers (compared to 48% on city level). However, different from other neighborhoods with serious littering problems, social cohesion was high: 62% of the residents indicated that they felt connected to the neighborhood (compared to 55% on city level). Treatment areas were all part of the same neighborhood, with comparable housing and population characteristics. No substantial differences between treatment areas were therefore expected. The combination of problem severity and high social cohesion made this neighborhood very suitable for an intervention targeting illegal littering by exploiting social commitments.

The municipality of Rotterdam took initiative in carrying out this experiment and consulted the Behavioral Insights Group Rotterdam (BIG’R^2^) on the experimental design and procedure. BIG’R consists of municipality employees and behavioral scientists from Erasmus University Rotterdam who collaborate to improve public policy. BIG’R is thus comparable in its aim and activities to the well-known BIT UK (John, 2014).

### Treatments

**FIGURE 2:**
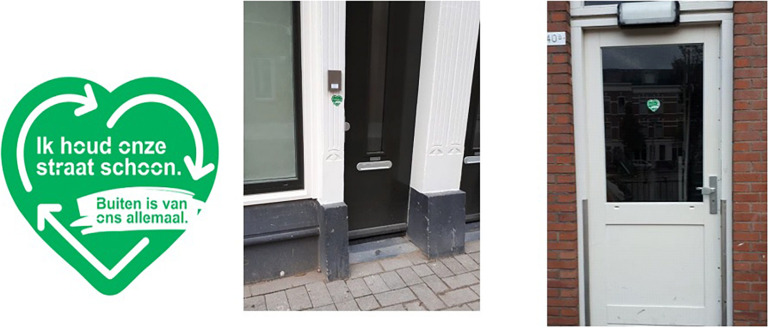
Commitment stickers on door(post): “*I keep our street clean – outdoors belongs to us all*.*”*

The nudging treatment encompassed two components: the door-to-door canvassing and the placement of reminder boards close to containers. The canvassing was carried out during a 2 week’s intervention period where, during 9 weekdays and one Saturday, five public information officers employed by the municipality reached 39% of households with maximum two attempts. Information officers were instructed to ask residents if they were familiar with the Rotterdam rules for garbage disposal, to explain them and inform households if necessary, and to provide households with a brochure summarizing rules and regulations for garbage disposal including some consequences of illegal garbage disposal. Importantly, only for the intervention treatment information officers also asked households to demonstrate commitment to keeping the neighborhood clean by placing a sticker (Figure 2) on or near their door or doorpost. With 74%, most of the reached households complied. In total, 29% of households in the treatment thus received the full treatment.

**FIGURE 3:**
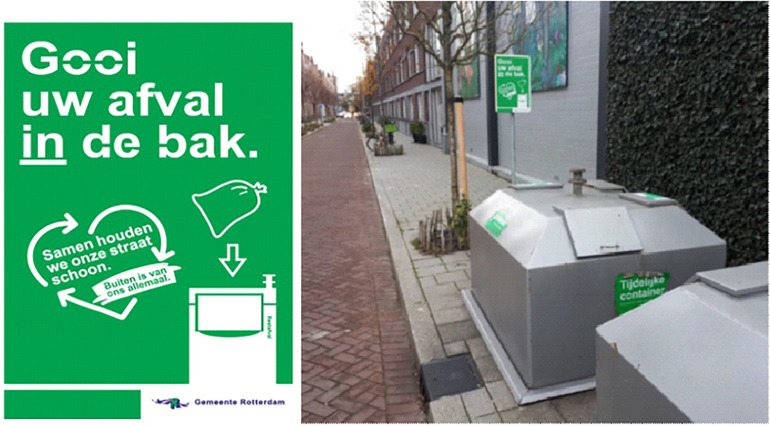
Reminder board next to containers: “*Throw your garbage in the container. Together we keep our street clean – outdoors belongs to us all*.*”*

From the second intervention week, information boards (Figure 3) were placed next to containers for the nudge treatment. These information boards remained in place at least until follow-up. Both the stickers and the boards emphasized shared responsibility for a clean neighborhood. Additionally, the board contained clear instructions for performing the desired behavior, both in written text and graphically. Due to practical reasons and municipality policy, the two nudges were thus integrated and overlapped in time making it impossible to evaluate them separately.

In the standard policy treatment, information officers conducted the same canvassing activities as in the intervention treatment. However, no commitment stickers and reminder boards were used. Note that for practical reasons the same information officers needed to conduct both canvassing treatments and could not be blinded to the different treatments. Public information officers reached 32% of households in this treatment. In the control area no actions regarding garbage disposal were carried out.

### Data Collection Procedure and Outcome Measures

**FIGURE 4:**
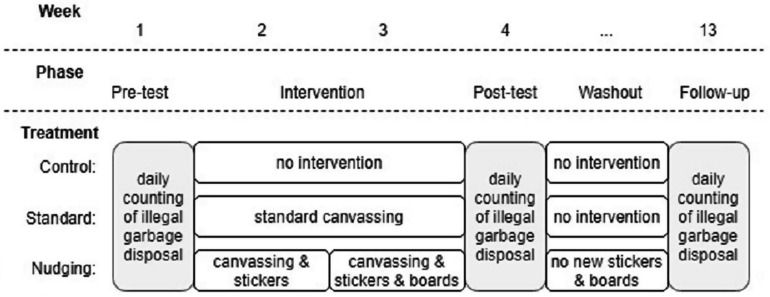
Research planning per treatment.

Garbage outside containers was measured during 1 week for each measurement. The pre-test measurement took place in week 1 (October 29 – November 3, 2019), the post-test measurement took place in week 4, and the follow-up measurement took place in week 13 (see Figure 4). This week was chosen for the follow-up because day length was comparable at that time to the post-test. Also, end of January was long enough after the holiday season, in which divergent garbage disposal could be expected (e.g., due to different working hours, and deviant production of garbage).

Only observational data on garbage disposals was collected and no personal data was registered. As a result, ethical review and approval as well as active consent from residents were not required for this study in accordance with local legislation and institutional requirements.

Illegal garbage disposals next to containers were recorded once a day by trained research assistants between 6:00 and 9:00 a.m. They registered if garbage was found outside each container (within a 5 m radius from the container), as well as the amount and type of garbage. Specifically, pictures were made, allowing researchers to check if new pieces of garbage had been placed or if it was remaining from previous days, i.e., garbage not picked up by the cleaning services of the municipality. Although the municipality tried to remove all displaced garbage daily early during the day, at some instances garbage was not picked up the same day. This was for example the case when it was hard to remove (e.g., a bucket chained to the container itself), or because of large items. At 11 instances (1.8% of all inspections) the same garbage was found a day later, and garbage registered the previous day was initially recorded as new illegally placed garbage. This was corrected in the data used for the analysis. Research assistants also checked and registered daily how the general treatment of containers was and if containers were full or hard to open. The order in which containers were checked varied each day. In the intervention area, research assistants also checked if the information boards were still in place and in good condition. If stickers were still visible and in place was not checked systematically, because information officers did not record which residents placed a sticker on their door (post). However, research assistants reported that stickers were still frequently present in the intervention area during follow-up.

As an outcome measure, we selected the number of days garbage was displaced near a container rather than the amount of displaced garbage. This approach was chosen because it was hard to quantify littering *behavior* by number, kind, or weight of items for example. Ten empty bottles are hardly comparable to one piece of furniture but may result from the exact same behavior of a single resident person. Thus, it was unclear if multiple items were the result of one or more littering instances from a single resident or multiple residents. Additionally, regardless of the number of items, additional cleaning needed to take place if any littering was detected. Using the number of days as an outcome variable was therefore also of high practical relevance. Therefore, the number of days that *new* placements were found was considered the main outcome variable.

### Data Analysis

Descriptive statistics from the pre-test were used to give an indication of baseline garbage disposals next to containers. Second, we compared the number of days per week on which garbage was illegally placed outside the containers between treatments: We used a mixed-design ANOVA with measurement time as a within-subjects variable, and treatment as a between-subjects variable. In case of significant main or interaction effects, repeated within-subjects contrasts for both treatment and time were applied to specify the results.

## Results

### Baseline Garbage Disposal

During the pre-test measurement, newly displaced garbage outside containers was found on average on 4.07 (*SD* = 1.82) days a week across treatments. Most garbage fell within the category of bulky waste (45.6% of all 1777 items), followed by paper (25.2%) and household waste (17.3%). In all three measurement periods, the highest proportion of containers with outside garbage was measured on Mondays (87%) and Sundays (55%), indicating that illegal garbage disposal was most common during the weekend.

### Effect of Treatment on Illegal Garbage Disposal

For the analysis, only containers for which there were no missing data were included (i.e., three containers of the commitment treatment were excluded because they were not accessible at follow-up). The assumption of sphericity was met [*Mauchly’s W*(2) = 0.859, *p* = 0.173]. There was no evidence for a main effect of the treatments on the number of days garbage was found outside containers [*F*(2, 24) = 0.22, *p* = 0.803], meaning that combined over the three measurement points the treatments were the same in the average number of days where illegal garbage was registered. However, the main effect of measurement time [*F* (2, 48) = 17.48, *p* < 0.001] was significant. Slopes differed between the pre-test and the post-test [*F* (2, 24) = 5.47, *p* = 0.012], but not between the post-test and follow-up [*F* (2, 24) = 2.27, *p* = 0.126], indicating that in general the treatments lead to a reduction in illegal garbage disposal directly after the intervention and remained stable until follow-up.

**FIGURE 5:**
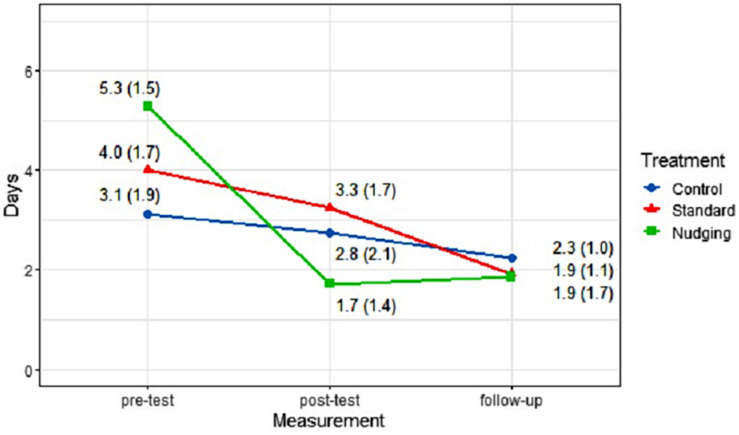
Mean (SD) number of days new garbage was found outside containers per treatment and measurement time.

As expected, the interaction between measurement time and treatment was significant [*F*(4, 48) = 3.78, *p* = 0.009; Figure 5], indicating that garbage disposals developed differently across treatments.

Inspecting treatments individually across measurement times, no differences between time points were detected for the control treatment [*F* (2, 14) = 0.82, *p* = 0.460]. Although a negative trend in the number of garbage disposals seemed to emerge, as expected, no significant decrease in illegal garbage disposals was found. However, the mean number of days that garbage was found outside containers differed between measurement times for the standard treatment [*F* (2, 22) = 10.52, *p* = 0.001] and the nudging treatment [*F* (2, 12) = 10.93, *p* = 0.002]: For the standard treatment this result was attributable to the difference between the post-test and follow-up [*F* (1, 11) = 1.94, *p* = 0.191; effect size: *d* = –0.84]. This means that no effect of the standard treatment was found directly after the intervention, while at follow-up a decrease was detectable. For the nudging treatment a decrease between pre-test and post-test (*F* (1, 6) = 0.05, *p* = 0.003; effect size: *d* = 2.40) was found, indicating that for this treatment results were observable right after the intervention and remained stable until follow-up.

When the mixed ANOVA is repeated including just the pre-test and the post-test, all container locations could be included in the analysis. This analysis confirmed the main results with a significant decrease of disposals found only for the nudging treatment [*F* (1, 9) = 33.92, *p* < 0.001]. When comparing only pre-test and follow-up, a significant decrease in disposals was found for the nudging treatment [*F* (1,6) = 21.13, *p* = 0.004], as well as the standard canvassing treatment [*F* (1, 11) = 17.60, *p* = 0.001], but not for the control treatment [*F*(1,7) = 1.49, *p* = 0.262].

## Discussion

We enriched the standard policy of the municipality of Rotterdam regarding illegal garbage disposals the “next to street containers” (i.e., door-to-door canvassing) with a combination of nudges that complemented already existing municipality policy because this approach was expected to be most effective (de Ridder et al., 2020). Specifically, we employed a commitment-nudge (e.g., King et al., 2013) and reminders with clear, explicit, and graphic instructions for the desired behavior (Kolodko et al., 2016). This approach was compared to the standard policy and a control treatment where not actions were taken to reduce illegal garbage disposal. At post-test, no effect of the standard policy or the control treatment was found. Adding nudges to the standard policy was highly effective at post-test, reducing the number of days garbage was illegally displaced with more than two-thirds and results remained stable until follow-up. This is an exceptionally large and lasting effect for a nudge intervention (Hummel and Maedche, 2019), with important consequences not just for municipality cleaning costs, but plausibly also for perceived neighborhood aesthetics (Roda et al., 2016) and perceived neighborhood satisfaction (Casakin and Reizer, 2017).

At follow-up, a decrease was also found in the area that received the standard policy and a decrease in illegal garbage disposals was found, resulting in comparable levels of illegal garbage disposal to the nudging treatment. For the nudging treatment and the control treatment, the effects remained stable at follow-up. The nudging treatment thus led to an immediate and lasting reduction in illegal garbage disposals whereas the standard policy led to a delayed and somewhat smaller reduction in illegal garbage disposals.

This delayed effectivity of the standard approach can be explained by the reach of the door-to-door canvassing campaign (32% of residents). Assuming that providing households with information brochures is an effective strategy to reduce illegal garbage disposals, still only those households that were reached could be expected to change their behavior directly after the intervention. Because no visible nudges were placed outside, other residents could not learn about the intervention other than by noticing a decrease in illegal garbage disposals. This decrease in garbage disposals could in turn signal a changing descriptive social norm (e.g., Cialdini, 2007; Sparkman and Walton, 2017), leading to less illegal displacements and littering by other residents (e.g., Kort et al., 2008), just as visible garbage invites more littering behavior (Keizer et al., 2011). Research shows that prosocial behavior can be contagious, especially when social proximity is high (Dimant, 2019), as it was in the studied area. The time needed for such self-reinforcing cycle of positive behavior change to unfold could explain both the lack of results at post-test as well as positive outcomes at follow-up in the standard canvassing treatment. However, an alternative explanation for the delayed effectivity of the standard policy could be that residents from the standard policy area learned over time from the adjacent neighborhood in which (visible) nudges were applied. Residents from the standard treatment area could have noticed both stickers and information boards, as well as the changed descriptive social norm in the nudging area, resulting in less littering in their own area. In general, the physical proximity of the three different treatment areas means that our results may be affected by contamination effects. Future studies can be conducted in more dispersed treatment areas; however, this might increase differences between areas.

Like all research, this study has its limitations. First, because both nudges were tested together, it remains unclear which nudge (or their combination) was responsible for the reduction in illegal garbage disposals. This can, however, be investigated in future research. Second, characteristics of the study area have implications for the generalizability of our findings: A large proportion of neighborhood residents had a non-western immigrant background (60%, as compared to 37% on city level; Neighborhood Profile Rotterdam, 2016). For a part of this group, language problems may have limited effectiveness of the intervention. Public administration officers involved in the canvassing campaign indeed indicated that they frequently encountered language barriers. Third, the study area was a neighborhood with relatively high levels of social cohesion. When conducted in neighborhoods with lower levels of social cohesion, the commitment and the community identity interventions may be less meaningful for the direct social environment and therefore less effective. Fourth, contextual factors that could not be influenced by the researchers (e.g., weather conditions) could have influenced dumping garbage disposal behavior. However, most likely, these treatments would have affected all areas equally since target neighborhoods were next to each other. Proximity of neighborhoods did, however, also come with a downside: We cannot rule out that effects found in the standard canvassing treatment were (partly) due to contamination effects from the adjacent intervention area. Passing regularly through a cleaner adjacent neighborhood may have strengthened the social norm and may thereby have reduced littering behavior. This effect might even have been strengthened by the canvassing itself, since this could have made the desired behavior more salient (Cialdini et al., 1990).

Lastly, between post-test and follow-up, three container locations for the nudging treatment were relocated outside of the research area, which could have influenced results because it complicated showing the desired behavior (i.e., disposing garbage in the containers). However, even under treatments possibly provoking illegal garbage disposals, because fewer containers were available, the effect of the nudging treatment remained stable.

Different from common nudging attempts to reduce littering that often involve enhanced visibility (e.g., colored bins, footsteps), our intervention was purposefully designed to complement existing measures enhancing their effectiveness at low cost. In addition, the employed nudges strengthened positive social norms that plausibly lead to more durable and robust effects than enhanced visibility (Lin et al., 2017). Yet, nudges in general have been criticized for being mere “fixes” that fail to challenge or change societal structures and patterns of behavior (Sellinger and Whyte, 2012; Whitehead et al., 2017). Following this line of reasoning, our approach did not address the underlying problem of (large amounts of) garbage being produced. Moreover, nudges were said to embrace a narrow definition of autonomy allowing experts to paternalistically program behavior (e.g., Hausman and Welch, 2010; John and Stoker, 2019). In fact, although the nudges were transparent (i.e., their intention was obvious to residents) residents were most likely not entirely aware of their working mechanism and how their behavior was intended to be changed. Yet, it comes as a strength of this research that residents were unaware of the research (i.e., a natural experiment), increasing ecological validity.

## Conclusion

Illegal garbage disposals were a persistent and serious problem in Rotterdam, resulting in high cleaning costs and decreased satisfaction with the neighborhood among residents. Enriching the standard canvassing policy of the municipality (i.e., door-to-door canvassing) with *nudges* that emphasized community identity and shared responsibility, evoked commitment, and provided reminders resulted in a two-third decrease of illegal garbage disposals when compared to the pre-test both at post-test and follow-up. This approach is thus highly promising in decreasing illegal garbage disposals to ultimately reduce cleaning costs, improve the aesthetical quality of urban areas, and reduce nuisance. In general, adding commitment strategies might be highly effective in improving canvassing at low cost. However, further testing with different neighborhoods is needed to judge the potential of this approach.

## Data Availability Statement

The raw data supporting the conclusions of this article will be made available by the authors, without undue reservation.

## Ethics Statement

Ethical review and approval was not required for the study on human participants in accordance with local legislation and institutional requirements. Written informed consent from the participants was not required to participate in this study in accordance with national legislation and the institutional requirements.

## Author Contributions

IM took the lead in designing the intervention and procedure. With Feedback from MD and SD, she was responsible for processing and analyzing the data as well as writing the first draft. IM and MD interacted with the municipality and made sure the experimental procedure was carried out correctly. MD and SD gave feedback and rewrote sections of this article, also adding literature. All authors contributed to the article and approved the submitted version.

## Conflict of Interest

The authors declare that the research was conducted in the absence of any commercial or financial relationships that could be construed as a potential conflict of interest.

## Publisher’s Note

All claims expressed in this article are solely those of the authors and do not necessarily represent those of their affiliated organizations, or those of the publisher, the editors and the reviewers. Any product that may be evaluated in this article, or claim that may be made by its manufacturer, is not guaranteed or endorsed by the publisher.

## Footnotes

1
https://www.cambridge.org/core/journals/behavioural-public-policy/article/when-and-why-defaults-influence-decisions-a-metaanalysis-of-default-effects/67AF6972CFB52698A60B6BD94B70C2C0

2
www.bigrotterdam.nl
